# Improvement of Adequate Digoxin Dosage: An Application of Machine Learning Approach

**DOI:** 10.1155/2018/3948245

**Published:** 2018-08-19

**Authors:** Ya-Han Hu, Chun-Tien Tai, Chih-Fong Tsai, Min-Wei Huang

**Affiliations:** ^1^Department of Information Management and Institute of Healthcare Information Management, National Chung Cheng University, Chiayi, Taiwan; ^2^Center for Innovative Research on Aging Society (CIRAS), National Chung Cheng University, Chiayi, Taiwan; ^3^Chiayi Chang Gung Memorial Hospital, Chiayi, Taiwan; ^4^Department of Information Management, National Central University, Taoyuan, Taiwan; ^5^School of Medicine, China Medical University, Taichung, Taiwan; ^6^Department of Psychiatry, Chiayi Branch, Taichung Veterans General Hospital, Chiayi, Taiwan

## Abstract

Digoxin is a high-alert medication because of its narrow therapeutic range and high drug-to-drug interactions (DDIs). Approximately 50% of digoxin toxicity cases are preventable, which motivated us to improve the treatment outcomes of digoxin. The objective of this study is to apply machine learning techniques to predict the appropriateness of initial digoxin dosage. A total of 307 inpatients who had their conditions treated with digoxin between 2004 and 2013 at a medical center in Taiwan were collected in the study. Ten independent variables, including demographic information, laboratory data, and whether the patients had CHF were also noted. A patient with serum digoxin concentration being controlled at 0.5–0.9 ng/mL after his/her initial digoxin dosage was defined as having an appropriate use of digoxin; otherwise, a patient was defined as having an inappropriate use of digoxin. Weka 3.7.3, an open source machine learning software, was adopted to develop prediction models. Six machine learning techniques were considered, including decision tree (C4.5), *k*-nearest neighbors (kNN), classification and regression tree (CART), randomForest (RF), multilayer perceptron (MLP), and logistic regression (LGR). In the non-DDI group, the area under ROC curve (AUC) of RF (0.912) was excellent, followed by that of MLP (0.813), CART (0.791), and C4.5 (0.784); the remaining classifiers performed poorly. For the DDI group, the AUC of RF (0.892) was the best, followed by CART (0.795), MLP (0.777), and C4.5 (0.774); the other classifiers' performances were less than ideal. The decision tree-based approaches and MLP exhibited markedly superior accuracy performance, regardless of DDI status. Although digoxin is a high-alert medication, its initial dose can be accurately determined by using data mining techniques such as decision tree-based and MLP approaches. Developing a dosage decision support system may serve as a supplementary tool for clinicians and also increase drug safety in clinical practice.

## 1. Introduction

Digoxin is the only oral heart medication approved by the United States Food and Drug Administration for use in enhancing positive inotropic effects and treating congestive heart failure (CHF). Because of its narrow therapeutic range and high drug-drug interactions (DDIs), digoxin is on the list of high-alert medications. Concentration changes of digoxin in the body are related to factors such as physiological characteristics, disease state, and coadministered drugs. Inappropriate dosages resulting in excessive drug concentrations in the body can cause numerous adverse reactions that affect the functioning of multiple organs [1, 2].

Digoxin toxicity is ranked fourth among adverse drug events (ADEs) involving older adults [3]. Digoxin also ranks among the top ten drugs requiring the interdisciplinary approaches of physicians, pharmacists, and nurses to jointly provide drug-related care [4]. Although the digoxin usage rate declined from 31.4% in 2001 to 23.5% in 2004, this trend was not reflected in the number of hospitalizations for digoxin toxicity, which indicates the difficulty involved in determining the appropriate dosage [5].

Digoxin-specific antibodies can be acutely administered to patients with digoxin toxicity; however, because adverse reactions occur directly in the heart or in the central nervous system, death may occur within a short time in severe cases. A previous study reported that when the serum digoxin concentration (SDC) exceeds 1.2 ng/mL, the mortality rate of patients who administered digoxin was 11.8% higher than that of patients in the placebo group [6].

Digoxin toxicity elevates mortality rates and increases medical costs. Gandhi et al. [7] indicated that the mean length of stay in the hospital as a result of digoxin toxicity was 3.3 ± 1.2 days, and the mean overall cost associated with digoxin toxicity was US$4087.05 ± US$2659.76. Moreover, these increased expenditures correlated significantly with increased SDC. According to reports in [1, 8], approximately 50% of digoxin toxicity cases are preventable, which motivated us to improve the treatment outcomes of digoxin, reduce the incidence rate of digoxin toxicity, and minimize the related medical costs.

Recent studies [9–17] on pharmacogenetics have investigated the influence of the ABCB1 gene polymorphism on SDC; however, the correlation between these two factors remains unclear, and conclusions have been inconsistent. Hoffmeyer et al. [11] reported an association of the ABCB1 gene polymorphism with SDC, and some investigators [12, 13, 16] confirmed the result of Hoffmeyer et al. [11]; on the other hand, Sakaeda et al. [15] contended that ABCB1 genotypes minimally influences digoxin pharmacokinetics, and Kurzawski et al. [17] noted that compared with other factors (i.e., age, diseases, herbs, and coadministered drugs), ABCB1 polymorphism slightly influences the digoxin level. In clinical practice, genetic testing is expensive and time-consuming, and its practical benefits in terms of determining an appropriate dosage remain unconfirmed. Therefore, using relatively inexpensive and accessible laboratory data to construct multiple prediction models is a feasible direction for future studies.

Recently, many researchers have applied statistical-based techniques to construct digoxin dosage prediction equations or models from clinical features. However, those studies considered relatively few variables and used tools that possessed poor prediction ability. Therefore, how to develop a robust digoxin dosage prediction model from clinical records is still a challenging task.

This study collaborated with a medical center in Taiwan and collected personal information of 307 inpatients who received digoxin treatment during the period of 2004–2013. Numerous studies have proven that machine learning techniques demonstrate superior performance on building prediction models. Therefore, a number of machine learning techniques, including decision tree (C4.5), *k*-nearest neighbors (kNN), classification and regression tree (CART), randomForest (RF), multilayer perceptron (MLP), and logistic regression (LGR), were used to construct prediction models for digoxin dosage adequacy. To evaluate the performance of the constructed classification systems, the accuracy, sensitivity, specificity, and the area under the ROC curve (AUC) of each classifier were assessed.

The paper is structured as follows: Section 2 presents the work related to digoxin dosage determination.  Section 3 provides the preparation of data, experimental setup, and performance measures. Sections 4 and 5 present thorough experimental results and discussions.  Section 6 concludes our study.

## 2. Related Work

In recent years, a number of methods have been proposed to predict digoxin dosage and treatment appropriateness (as shown in Table 1). These methods are largely statistical-based techniques, such as regression model, Konishi equation, and the pharmacokinetics-based nonlinear mixed-effect modeling (NONMEM). For example, the NONMEM is a widely implemented method that enables physicians to estimate the appropriate dosage of a drug by calculating the SDC or clearance rate. When establishing a pharmacokinetics model, one must possess a complete understanding of various statistical models, including parameters related to pharmacokinetics structural models. However, Tolle et al. [37] argued that the performance of pharmacokinetic modeling can be significantly affected by population characteristics of patients and many other interference factors. In addition, the pharmacological properties of digoxin are more complex than those of other drugs, and its parameters are not easily established. As a result, developing a statistical-based model to digoxin dosage prediction is highly difficult.

Few recent studies have applied machine learning techniques to digoxin treatment decisions. For example, Albert et al. [8] applied artificial neural network (ANN) to predict toxicity after digoxin administration, and results showed the performance of the neural network model better than the logistic regressions one in both sensitivity and specificity. Martín et al. [24] also adopted ANN to predict digoxin toxicity and reported similar outcomes. However, ANN-based methods have poor interpretability. Clinicians cannot easily understand the output generated by ANN, which limit its clinical applications.

Previous studies reveal that considering SDC or the clearance of digoxin (CL) provides promising prediction results. In clinical practice, physicians normally test patients' SDC, and adjust medication dose according to therapeutic drug monitoring (TDM) and personal experience. However, for patients receiving such medications for the first time, physicians are unable to perform dose evaluation via SDC or CL. Therefore, our primary goal of this study was to apply machine learning techniques to propose an alternative approach that could facilitate the prediction of the initial dosage adequacy of digoxin.

In addition, previous studies utilized different population of ethnic groups to develop various prediction models, which reveals that ethnicity is an important factor in digoxin dosing. To the best of our knowledge, this study first adopts machine learning techniques to construct digoxin dosage prediction models for the Chinese ethnic group; our results can provide significant information in clinical decision support.

Our main contribution of this work is to construct more practical and robust prediction systems, including all drugs having DDI when combined with digoxin is indispensable. The research results indicated that using a high-performance classification model can effectively improve the prediction accuracy rate, thereby confirming the value of this technique in clinical applications.

## 3. Materials and Methods

### 3.1. Data

Research data were obtained through a medical records database at a medical center in Taiwan; specifically, the medical records were of patients who had been hospitalized and had their conditions treated with digoxin between 2004 and 2013. In addition, because some of the patients may have taken digoxin prior to hospitalization, a 3-month washout period was imposed to ensure the accuracy of the prediction models after considering the drug's half-life (i.e., 36 to 48 hours with normal renal function) [38]. Therefore, patients who had consumed digoxin within 3 months prior to hospitalization were excluded from this study. The Chang Gung Memorial Hospital Institutional Review Board approved the study protocol (105-0542C). Written consent from the study was deemed unnecessary because the dataset comprises only deidentified secondary data for research purposes, and the Chang Gung Memorial Hospital Institutional Review Board issued a formal written waiver of the need for consent and approved the study.

In addition, the medical records indicated that most of the patients had been prescribed half a digoxin pill per day (i.e., 0.125 mg daily) as an initial dosage. To obtain more reliable results, therefore, this study only selected the patients who had been prescribed 0.125 mg daily as the study samples.

To construct the prediction models, each patient was classified based on whether the treatment was considered to be appropriate or inappropriate (i.e., dependent variable). Currently, SDC is the primary reference indicator for determining the appropriate dosage of digoxin; if the SDC is controlled at 0.5–0.9 ng/mL, this can effectively reduce all-cause mortality and hospitalization rates [2, 20]. Therefore, a patient with SDC being controlled at 0.5–0.9 ng/mL after his/her initial digoxin dosage was defined as having an appropriate use of digoxin; otherwise, a patient was defined as having an inappropriate use of digoxin.

Each hospitalization record contained 10 independent variables, including demographic information (i.e., sex, age, and weight) and test data (i.e., alanine aminotransferase (ALT), aspartate aminotransferase (AST), serum creatinine concentrations (SCr), blood urea nitrogen (BUN), albumin (ALB), and serum (K^+^)). Whether the patients had any specific illnesses (CHF) was also noted.

Furthermore, DDI was another critical factor on digoxin dosage determination and required consideration before constructing the prediction models because it can affect SDC performance and threaten the patient's life in severe cases [1, 2]. Therefore, we referenced DDI information released by the Ministry of Health and Welfare in Taiwan and compared this information with records from the drug registry used at the studied medical center, subsequently identifying 26 drugs that are known to produce major DDIs when coadministered with digoxin (Table 2). The risk of DDI was considered for the patients who were prescribed any of the drugs listed in Table 2 while undergoing digoxin therapy, and the data observations were accordingly categorized as “with DDIs” or “without DDIs” before conducting the analysis.

To construct highly reliable digoxin dosage prediction models, the collected 307 clinical cases were then further divided into two datasets: one containing 222 cases with DDIs (i.e., DDI group) and the other containing 85 cases without DDI (i.e., non-DDI group).

### 3.2. Experimental Setup

To develop prediction models for evaluating the appropriateness of initial dosage of digoxin, this study adopted Weka 3.7.3 (www.cs.waikato.ac.nz/ml/weka), an open source machine learning software. A number of machine learning techniques were considered, including decision tree (C4.5), *k*-nearest neighbors (kNN), classification and regression tree (CART), randomForest (RF), multilayer perceptron (MLP), and logistic regression (LGR). In addition, the prediction performance of machine learning techniques can be significantly influenced by the internal parameter setting. To optimize the prediction performance of the selected techniques, the CVParameterSelection metalearner module implemented in Weka was used. In this module, we first selected a prediction technique and specified various parameter combinations. The algorithm then automatically searched the optimal parameter setting based on the best prediction results using cross validation. The parameter settings used in this study is listed in Table 3.

Previous study showed that the class imbalance problem can significantly affect the learning performance [39]. To improve the classification performance, a resample module in Weka is adopted to modify the distribution of instances of two classes. Specifically, the distribution of the class label is modified to be almost identical by oversampling the inadequate class and undersampling the adequate class. In addition, the random resample technique is applied thirty times to construct datasets; for each generated dataset, ten-fold cross validation is then applied in all the experimental evaluations [40]. Specifically, each dataset is partitioned into ten complementary subsets, wherein any nine were used for model training, and the remaining subset was used for model testing.

### 3.3. Performance Measures

To evaluate the performance of the constructed classification systems (i.e., prediction models), the accuracy, sensitivity, and specificity of each classifier was assessed. These were measured using a confusion matrix, as shown in Table 4.

The prediction accuracy, sensitivity, and specificity were obtained using the following formulas:

(1) $$\begin{array}{c} \text{Accuracy}=\frac{a+d}{a+b+c+d},\\ \text{Sensitivity}=\frac{a}{a+b},\\ \text{Specificity}=\frac{d}{c+d}. \end{array}$$

In addition to use the performance measures, the receiver operating characteristic (ROC) curve is plotted, and the area under the ROC curve (AUC) is also calculated. The ROC curve can illustrate the performance of a binary classifier as its discrimination threshold is varied. It can be generated by plotting the sensitivity against (1−specificity) at different discrimination threshold settings. When using normalized units, the AUC is equal to the probability that a classifier will rank a randomly chosen positive (i.e., adequate digoxin dosage) sample higher than a randomly chosen negative (i.e., inadequate digoxin dosage) one. Hosmer and Lemeshow [41] provide general rules to categorize the evaluation performance using the AUC as “excellent” if AUC ≥ 0.9, “good” if 0.9 > AUC ≥ 0.8, “fair” if 0.8 > AUC ≥ 0.7, “poor” if 0.7 > AUC ≥ 0.6, and “`very poor” if AUC < 0.6.

## 4. Results

Table 5 lists variables and descriptive statistics of the non-DDI and DDI groups. As mentioned earlier, there were 222 inpatient cases drawn from the group with DDIs and 85 cases without DDIs, resulting in 307 valid clinical cases in total.

Table 6 shows the experimental results of the non-DDI and DDI groups, which were analyzed using 6 specific classification techniques. Sensitivity, specificity, accuracy, and area under the curve (AUC) were employed to evaluate the effectiveness of the prediction models. To facilitate result interpretation, the means and standard deviations of the above four metrics from 30 datasets are listed in the table.

Regarding the accuracy of the prediction models for the non-DDI group, the RF result was the most favorable (83.9%), followed by the MLP result (80.9%), and CART and C4.5 yielded accuracy rates above 75%. The remaining classifiers performed poorly, all of which yielded accuracy rates below 65%. Because of its complex medication regimens, the DDI group generally exhibited prediction accuracies that were lower than those of the non-DDI group. However, for the DDI group, the RF (80.5%) continued to yield the most favorable result (80.5%), followed by the MLP, CART, and C4.5. The other classifiers performed poorly, yielding no result higher than 60.2%. Overall, the decision tree-based approaches (i.e., RF, C4.5, and CART) and MLP exhibited markedly superior accuracy performance, regardless of DDI status.

The AUC performance values were distributed in the 0.533–0.912 range. In the non-DDI group, RF was excellent, followed by MLP, CART and C4.5; the remaining classifiers performed poorly. For the DDI group, the RF performance was the best (0.892; good), followed by CART, MLP, and C4.5; the other classifiers' performances were less than ideal.

Finally, a comprehensive assessment of the various indicators revealed that, regardless of DDI status, the decision tree-based classifiers clearly outperformed the kNN and LGR classifiers, demonstrating the superior accuracy of the decision tree-based approaches for predicting appropriate dosages.

## 5. Discussion

The safety of using high-alert medications such as digoxin is a pressing topic [1, 2, 8]. Previous studies have employed statistical models [1, 2, 20, 22, 25] and pharmacokinetics [18, 21, 23, 26, 28, 29, 31–36], and data mining and machine learning techniques have only recently been adopted to improve model predictability [8, 24]. This study investigated decision tree-based approaches, which were identified to exhibit an average performance superior to that of other techniques. In addition, the information obtained through decision tree-based approaches can be presented as if–then rules that physicians can refer to determine the appropriate dosage of digoxin to prescribe to patients.

Among the investigated three decision tree-based approaches, an RF classifier exhibited the optimal prediction performance. RF is an ensemble classifier that combines bagging and decision tree techniques. Let *m* be the number of variables and *n* be the number of instances, the time complexity for constructing an unpruned decision tree is *O*(*m* · *n* · log(*n*)). In building RF classifier, one should define the number of bootstrap sample sets (denoted as nbt) and the number of variables that can be randomly selected for each sample set (denoted as nvar). Therefore, the complexity of building an RF classifier is *O*(nbt · nvar · *n* · log(*n*)).

In addition to comparing the predictive capabilities of various classification techniques, this study further evaluated the importance of multiple variables to provide a reference for clinicians. As shown in Table 5, 10 variables were used in analysis, which is more than the number of variables examined in previous studies. Furthermore, previous studies have evaluated only 8 drug types for DDIs; in this study, a total of 26 drugs with major DDI effects were included.

By calculating gain ratios, we found that the most crucial variables influencing dosage appropriateness were in the order SCr, serum K^+^, CHF, and DDI (as shown in Table 7). Following a discussion with the physicians and pharmacists at the case medical center, the validity of the aforementioned results is described as follows.

First, SCr is a major indicator of renal function. Because digoxin in the body is mainly excreted through the kidneys, poor renal function may result in a longer half-life of digoxin in the body and an elevated SDC, thereby inducing digoxin toxicity. We presented that renal function plays a significant role for the SDC, and the finding is consistent with the results of earlier studies about digoxin doses in patients with renal failure [42–45]. Therefore, clinicians should consider prescribing a low dosage of digoxin to patients with poor renal function. Furthermore, our study indicated that serum K^+^ is also a critical factor of dosage appropriateness. Digoxin's primary mechanism of action involves inhibition of the sodium potassium adenosine triphosphatase (Na+/K+ ATPase), mainly in the myocardium. Because potassium and digoxin compete for the same ATPase-binding site, excessively low concentrations of potassium ions in the body cause the cardiomyocytes to absorb additional digoxin, thereby increasing toxicity risk [46–50]. Therefore, clinicians should monitor their patients' electrolyte levels (particularly serum K^+^) to facilitate dosage adjustment. Second, one of the major indications for using digoxin is CHF, and accumulative evidences indicate that low-dose digoxin can reduce hospitalization and mortality in patients with heart failure [51–53]. However, a history of CHF may influence the metabolism of digoxin. Previous studies showed that digoxin absorption is slower, and peak concentration is lower in patients with CHF than in healthy volunteers due to reduced gastrointestinal motility, congestion in the gut wall, and reduced splanchnic blood flow in patients with CHF [54–56], thereby causing changes of SDC. Finally, DDI involving high-alert medications has gained considerable attention in the field of medicine, particularly digoxin, which has a narrow therapeutic concentration range [2, 8, 18, 20, 23, 24, 27–31, 34–36]. DDI involving digoxin can easily lead to an elevated SDC and subsequent toxicity reaction [57]. Although this study included only drugs that are known to produce major DDI with digoxin, the results are sufficient for confirming the importance of DDI. The results also confirmed that adequate laboratory information can effectively assist physicians in determining the appropriate dosage.

## 6. Conclusion

Medication safety has received considerable attention in the medical community in recent years. It is particularly crucial for high-alert medications that have a narrow therapeutic range or easily induce toxicity. Digoxin is a high-alert medication, and prescribing an inappropriate dosage of digoxin can easily cause severe side effects and even fatality. The objective of this study was to predict the appropriateness of the initial dosage of digoxin. Six classification techniques were adopted to establish multiple classification models for predicting dosage appropriateness. The medical records of 307 hospitalized patients were used to confirm that the prediction accuracy rates of all adopted techniques exceeded those of the physicians in the actual patient cases. Overall, the RF prediction model exhibited the optimal effectiveness; the decision tree-based approaches exhibited favorable performance and can be used by clinicians as aid for making clinical dosage decisions.

Although digoxin is characterized by complex pharmacological properties, this study confirmed that the adequate use of laboratory data and consideration of numerous variables can yield favorable prediction effectiveness. In conjunction with clinical experience, the suggested prediction models can facilitate clinicians making a proper decision in practice. The improvement in the safe use of digoxin will be of benefit to both clinicians and patients.

Some limitations of the present study should be addressed because they may restrict generalizability and are indicative of the need for further research. First, the data used in this study were collected from a single medical institution. Proceeding with the evaluations of clinical cases from other hospitals is critical for confirming the validity of the model. Second, other potentially valuable features, such as data collected from the nursing information system and clinical pathway, can be considered for use in the model. Finally, because most of the inpatients took half a digoxin pill per day as an initial dosage in the case hospital, this study only focused on evaluating the adequacy of prescribing 0.125 mg digoxin as an initial dosage for the inpatients daily. Future studies may directly predict adequate digoxin dosage when a large enough number of samples are collected.

## Figures and Tables

**Table 1:** Recent studies on digoxin dosage prediction.

Study	Sample size	Study population	*Y*	Method	Demographic data (gender,age, TBW^*∗*^)	SCr^*∗*^	ALT/AST^*∗*^	BUN^*∗*^	ALB^*∗*^	K^+^^*∗*^	Dose^*∗*^	SDC^*∗*^	CHF^*∗*^	DDI^*∗*^	Num. of variables
Albert et al. [8]	125	Spain	Toxicity	(1) logistic regression (2) neural networks	V	V				V	V	V	V	V(5)^*∗∗*^	7
Bauman et al. [2]	54	Caucasians (America)	SDC^*∗*^	Multiple linear regression	V	V					V	V		V(4)^*∗∗*^	5
Chen et al. [18]	142	China	CL^*∗*^	NONMEM	V	V	V	V			V	V	V	V(4)^*∗∗*^	8
Jiratham-Opas et al. [19]	114	Thailand	SDC^*∗*^	Konishi equation	V	V					V	V	V	V(9)^*∗∗*^	6
Kockova et al. [20]	222	Czech Republic	Toxicity	(1) Fisher's exact test (2) logistic regression	V	V				V	V	V		V(6)^*∗∗*^	6
Komatsu et al. [21]	192	Japanese	SDC^*∗*^	NONMEM	V	V					V	V	V	V(11)^*∗∗*^	6
Konishi et al. [22]	235	Japanese	SDC^*∗*^	Hyperbolic regression model	V	V					V	V	V		5
Kroese et al. [23]	45	Caucasians (UK)	SDC^*∗*^	PharmDIS	V	V					V			V(3)^*∗∗*^	4
Martín et al. [24]	257	Spain	Toxicity	Neural networks	V	V					V			V(1)^*∗∗*^	4
Martin-Suarez et al. [25]	63	Spain	Dose	Linear regression	V	V		V			V	V			5
Martin-Suarez et al. [26]	8	Spain	Dose	NONMEM	V	V					V	V			4
Muzzarelli et al. [27]	40	Switzerland	Dose	Konishi equation	V	V					V		V	V(8)^*∗∗*^	5
Petcharattana [28]	130	Thailand	SDC^*∗*^	NONMEM	V	V					V	V	V	V(6)^*∗∗*^	6
Suematsu et al. [29]	172	Japanese	CL^*∗*^	NONMEM	V	V					V	V	V	V(1)^*∗∗*^	6
Suematsu et al. [30]	181	Japanese	CL^*∗*^	Least-squares analysis	V	V					V	V	V	V(1)^*∗∗*^	6
Yukawa et al. [31]	94	Japanese	Dose	NONMEM	V	V					V	V	V	V(1)^*∗∗*^	6
Yukawa et al. [32]	117	Japanese	CL^*∗*^	NONMEM	V	V					V	V	V		5
Yukawa et al. [33]	71	Japanese	SDC^*∗*^	NONMEM	V	V					V	V			4
Yukawa et al. [34]	106	Japanese	CL^*∗*^	NONMEM	V	V					V	V		V(2)^*∗∗*^	5
Yukawa et al. [35]	385	Japanese	CL^*∗*^	NONMEM	V	V					V	V	V	V(1)^*∗∗*^	6
Zhou et al. [36]	119	China	SDC^*∗*^	NONMEM	V	V	V	V	V		V	V	V	V(4)^*∗∗*^	9
Our study	307	Taiwan	Dose adequacy	Machine learning methods	V	V	V	V	V	V	V	V	V	V(26)^*∗∗*^	10

^*∗*^ALB, albumin; ALT/AST, alanine aminotransferase/aspartate aminotransferase; BUN, blood urea nitrogen; CHF, congestive heart failure; CL, the clearance of digoxin; DDIs, drug-drug interactions; Dose, digoxin daily dose; K^+^, serum potassium; SCr, serum creatinine; SDC, serum digoxin concentration. ^*∗∗*^The number in the parentheses represents the number of DDI drugs considered in the study.

**Table 2:** A list of medicines causing major DDI when combined with digoxin.

Amiodarone	Dronedarone	Norepinephrine	Spironolactone
Alprazolam	Epinephrine	Oxytetracycline	Succinylcholine
Boceprevir	Erythromycin	Propafenone	Tetracycline
Calcium carbonate	Indomethacin	Propantheline	Thiazide diuretics
Clarithromycin	Itraconazole	Quinidine	Verapamil
Dopamine	Mifepristone	Ritonavir	
Doxycycline	Minocycline	Saquinavir	

**Table 3:** Parameter settings in WEKA.

Method	Parameters	Value/Range	Best parameter setting
J48	Confidence factor	0.1–0.5	0.25
	Minimum number of instances per leaf	2–50	2
IBk	Number of neighbors	2–10	2
SimpleCART	Minimum number of instances per leaf	2–50	2
RandomForest	Number of trees	5–10	10
	Number of attributes to be used in random selection	2–8	4
Multilayer perceptron	Number of hidden nodes	3–14	7
	Learning rate	0.1–0.6	0.3
	Momentum factor	0–0.9	0.2
	Maximum number of epochs	300–1000	500
AdaBoostM1	Number of iterations	10	10
	Weight threshold for pruning	100	100

**Table 4:** Confusion matrix.

		Predicted class	
		Adequate	Inadequate
Actual class	Adequate	*a*	*b*
	Inadequate	*c*	*d*

**Table 5:** Summary statistics for the non-DDI and DDI groups.

Variable	Non-DDI group		DDI group	
	Range	Summary statistics	Range	Summary statistics
Gender	Male/female	Male: 40	Male/female	Male: 121
		Female: 45		Female: 101
Age (years)	38 to 94	*μ* = 73.95, *σ* = 10.53	23 to 101	*μ* = 74.84, *σ* = 11.28
Weight (kg)	35 to 89	*μ* = 57.37, *σ* = 10.62	33 to 105	*μ* = 55.95, *σ* = 12.90
SDC	0.2 to 2.4	*μ* = 0.628, *σ* = 0.470	0.2 to 4.3	*μ* = 0.85, *σ* = 0.59
ALT	8 to 475	*μ* = 46.05, *σ* = 81.74	5 to 1381	*μ* = 85.26, *σ* = 191.95
AST	17 to 1776	*μ* = 169.69, *σ* = 437.21	12 to 2615	*μ* = 105.77, *σ* = 290.97
SCr	0.38 to 3.55	*μ* = 1.046, *σ* = 0.653	0.29 to 12.1	*μ* = 1.361, *σ* = 1.270
BUN	1.9 to 83	*μ* = 21.43, *σ* = 14.50	2 to 219	*μ* = 35.49, *σ* = 30.10
ALB	1.4 to 4.2	*μ* = 2.913, *σ* = 0.597	1.1 to 4.2	*μ* = 2.546, *σ* = 0.611
K+	2.5 to 5.4	*μ* = 3.96, *σ* = 0.614	2.69 to 6.80	*μ* = 3.979, *σ* = 0.723
CHF	Yes/no	Yes: 35/no: 50	Yes/no	Yes: 132/no: 90

**Table 6:** Performance evaluation of the classifiers for the non-DDI and DDI groups.

Group	Method	Sensitivity	Specificity	Accuracy	AUC
Non-DDI	C4.5	0.705/0.091	0.806/0.078	0.759/0.061	0.784/0.065
	CART	0.696/0.095	0.825/0.067	0.765/0.055	0.791/0.057
	RF	0.782/0.090	0.888/0.054	0 0.839/0.041	0.912/0.032
	kNN	0.619/0.172	0.547/0.162	0.592/0.068	0.606/0.070
	LGR	0.566/0.145	0.715/0.117	0.648/0.078	0.661/0.097
	MLP	0.741/0.091	0.871/0.057	0.809/0.059	0.813/0.071

DDI	C4.5	0.701/0.060	0.759/0.050	0.732/0.029	0.774/0.030
	CART	0.728/0.051	0.776/0.050	0.754/0.031	0.795/0.031
	RF	0.790/0.050	0.817/0.043	0.805/0.027	0.892/0.020
	kNN	0.545/0.094	0.651/0.087	0.602/0.042	0.634/0.048
	LGR	0.464/0.139	0.621/0.145	0.551/0.042	0.556/0.058
	MLP	0.745/0.058	0.799/0.042	0.774/0.037	0.777/0.051

**Table 7:** Ranking of selected variables by gain ratio.

Rank	Variable	Gain ratios
1	SCr	0.0826
2	Serum K^+^	0.0428
3	CHF	0.0129
4	DDI	0.0101

## Data Availability

The data used to support the findings of this study are available from the corresponding author upon request.
